# Concerning Some Uses of Oxy-Chloride of Zinc

**Published:** 1887-03

**Authors:** N. S. Jenkins

**Affiliations:** Dresden, Germany


### CONCERNING SOME USES OF OXY-CHLORIDE OF ZINC.

READ BEFORE THE AMERICAN DENTAL SOCIETY OF EUROPE.

BY N. S. JENKINS. D. D. S., DRESDEN, GERMANY.

If one is to make any use of this material, whose bad qualities are at first more noticeable than its good qualities, it is especially desirable to have a good article. For my purposes I have been much pleased with the compound formed by uniting Poulsen's oxide powder No. 1, such as is furnished for phosphate fillings, with liquid chloride. It hardens rapidly, adheres to the walls with great tenacity, possesses a clear white color, and, when kept dry for sufficient time, attains a mechanical hardness nearly equal to the best phosphate.

Of course we all make occasional use of oxy-chloride of zinc, with a proper degree of mistrust, for purely temporary operations, and when we can control the patient, accomplish our purposes with great accuracy. But my object in this paper is to explain the use I make of oxy-chloride of zinc in permanent operations.

First, in filling over exposed pulps.

Those of us who begin to number ourselves among the older practitioners, recollect with bitterness our grevious disappointment when we first placed our reliance upon this painful escharotic as a material for capping pulps. How many failures of our own and of our most skillful colleagues were necessary before we could quite resign faith in a substance whose antiseptic quality and plastic character, making the most delicate manipulation and adjustment possible, promised such good results. I use oxy-chloride of zinc still, but I never let it touch the pulp. When the cavity has been prepared and slightly moistened with creosote, I place a piece of dry paper, such as is used in books of gold foil, over the pulp, taking care to cut the paper somewhat larger than the exposure, and then pack oxy-chloride quickly and gently over it, generally completely filling the cavity. The object of using dry paper is to be able to see, as one can do before it is made transparent through wetting with creosote, exactly where the paper is placed. Besides, a bit of dry paper absorbs any slight excess of creosote. So soon as the filling is properly hardened, the cavity is reshaped, taking then such a form as may seem desirable, it being quite possible in case of necessity to make reliable retaining points in the oxy-chloride. It is generally best to leave a considerable portion of this material in the cavity, but so much of it should be cut away from the edges that the permanent filling, which is now packed over it, protects it perfectly from exposure to the fluids of the mouth.

I quite understand that many dentists feel great confidence in capping with some of the non-irritating cements; but I prefer the method above mentioned, because it places a thin, uniform, non-conducting, non-irritating substance over the pulp, which it protects from pressure, and is itself then covered with an antiseptic, bacteria-destroying cement, which neither expands nor contracts after crystallization, and which simulates in density the dentine, which binds together and efficiently supports weak walls, retains its color, and supplies a solid base, making the permanent filling of the cavity possible at a single sitting. Moreover, this method is easier to me than capping with a non-irritating cement, and covering that with another cement; and, last of all, I have found it uniformly successful.

Secondly, in many large cavities where the pulp is not exposed, and especially where the dentine is soft and the patient cannot be kept under proper observation, where perhaps under ordinary circumstances a temporary operation would be indicated, I first fill with oxy-chloride, and then immediately cover with gold. Much time can often be saved by this method, but its principal advantages are comparative non-conductibility, antiseptic quality, a union with the walls of the cavity (not simply an adaptation, such as we obtain with gold), and consequent strengthening of weak walls, with perfect preservation of color. In those rare cases where amalgam is indicated, cases which tend to become always rarer as skill increases and gold improves, this method is especially valuable. If with the aid of well-arranged retaining points and grooves it is possible to cap oxy-chloride with a thin wall of amalgam and then leave the dam in position until crystallization has taken place, there is practically no danger of shrinkage nor of discoloration.

The pain which results from applying oxy-chloride to sensitive dentine can be at once subdued by holding whisky, or any other distilled liquor, in the mouth for a few minutes.

Thirdly, oxy-chloride is invaluable to me for filling pulp canals. I prefer to fill the extreme point with a minute shred of cotton, as pain is apt to follow the application of oxy-chloride to the foramen. When all the preliminary steps have been taken, with that attention to detail so indispensable to success, such as securing direct access to the roots, obtaining perfect dryness, good walls, complete disinfection and removal of all debris, I fill the canals with creosote by means of a filament of cotton on a very fine barbed broach. Then I carry on a carefully selected Donnalson broach a bit of dry cotton through that creosote to the foramen, and pack it securely there. This is an operation which requires, in such places as the buccal roots of a superior molar, a steadiness of hand and a delicacy of touch of the highest order. It is no easy matter to wind an almost microscopic shred of cotton about the point of a flexible broach so that it can be carried to the point of a tortuous canal; but this operation is facilitated by allowing the cotton to become saturated with creosote only after entering the canal. In many cases it is desirable and necessary to enlarge the pulp canals, but this is occasionally impossible, and it is in these cases that great manipulative skill is called for.

After the foramen has been stopped in the manner above mentioned, the surplus creosote is absorbed. Cotton saturated with thin oxy-chloride is then carried quickly on broaches, a considerable number of which have been previously prepared by winding cotton about the points, so that no time shall be lost, as the oxy-chloride rapidly sets, until the roots are completely filled. Then the crown is also filled with oxy-chloride, the surplus cut away and the operation completed with gold.

This is to me the most rapid as well as the most satisfactory method of filling devitalized teeth, and by means of which I am able to secure the maximum of strength and density with the minimum of pressure, together with security against subsequent discoloration.

I do not offer these observations as suggesting new methods of practice, but only to present some of my methods of manipulation in daily use, in the hope that discussion may develop points of practical utility. We all have methods differing in some particulars from those of all other men, and a knowledge of these various methods cannot fail to be sometimes useful.

